# Nasal Septal Abscess Causing Near-Fatal Toxic Shock Syndrome With Multiorgan Dysfunction: A Case Report

**DOI:** 10.7759/cureus.99885

**Published:** 2025-12-22

**Authors:** Bakri Alali, Nissar Shaikh, Maher Abu Sunbol, Mahmoud Alshabani

**Affiliations:** 1 Surgical Intensive Care, Hamad Medical Corporation, Doha, QAT; 2 Medical Education, Hamad Medical Corporation, Doha, QAT

**Keywords:** acute kidney injury, acute respiratory distress syndrome, bacteremia, methicillin-resistant staphylococcus aureus, nasal septal abscess, necrotizing pneumonia, periorbital cellulitis, pneumothorax, toxic shock syndrome

## Abstract

Nasal septal abscess (NSA) is a rare collection of pus between the nasal septum and the surrounding lining tissues. We report a case of methicillin-resistant *Staphylococcus aureus* (MRSA) NSA rapidly progressing to bacteremia, toxic shock syndrome (TSS), multiple organ dysfunction syndrome (MODS), and necrotizing pneumonia within days of presentation, with a favorable outcome. A young male diagnosed with NSA, periorbital cellulitis, and bilateral cavernous sinus thrombosis was admitted to the ICU. His condition deteriorated further, developing septic shock and requiring endotracheal intubation and two vasopressors (noradrenaline and vasopressin). He also developed acute kidney injury, necessitating continuous renal replacement therapy. The NSA was drained, and blood and pus cultures grew MRSA. Antibiotics were adjusted to meropenem, vancomycin, and clindamycin. On day 4, the patient developed severe acute respiratory distress syndrome, requiring prone positioning. After receiving a course of IVIG, his condition began to improve. His ventilation/perfusion (P/F) ratio improved, urine output normalized, and vasopressors were weaned by day 6. He subsequently developed necrotizing pneumonia that led to tension pneumothorax during ventilator weaning, which was managed with chest drain insertion. The patient underwent tracheostomy and was eventually weaned from mechanical ventilation, transferred to the medical ward, decannulated, and discharged home. MRSA NSA can progress to life-threatening TSS with MODS and necrotizing pneumonia, which may complicate ventilator weaning with tension pneumothorax. Early recognition and aggressive multidisciplinary management are critical for favorable outcomes.

## Introduction

Nasal septal abscess (NSA) is a rare clinical entity, most commonly caused by trauma or nasal septal hematoma [1]. In adults, NSA has been infrequently reported as resulting from habitual nose picking [2]. It occurs more frequently in children and males [1]. Methicillin-sensitive *Staphylococcus aureus *(MSSA) and methicillin-resistant *S. aureus *(MRSA) are the most common causal organisms in immunocompetent, otherwise healthy patients. Rarely, NSA has been reported in an immunocompromised patient due to amoebiasis [3].

Complications of NSA can include facio-orbital cellulitis, pansinusitis, meningitis, brain abscess, and cavernous sinus thrombosis [4]. Additionally, NSA can lead to septal necrosis and subsequent saddle nose deformity [2,4]. To date, progression of NSA to MRSA bacteremia, toxic shock syndrome (TSS), and multiple organ dysfunction syndrome (MODS) has not been reported. Here, we describe a case of MRSA NSA progressing to bacteremia, TSS, MODS, and necrotizing pneumonia, which resulted in a favorable outcome.

## Case presentation

A previously healthy 44-year-old male presented to the ED with nasal pain and swelling for two days and right orbital swelling for one day, along with a history of fever for three days. He reported a preceding one-week history of low-grade fever and rhinorrhea, for which he had received treatment at a primary health center. Further history revealed habitual nasal picking.

On examination, he was alert and hemodynamically stable (heart rate 70 beats/min; blood pressure 143/60 mmHg) with oxygen saturation of 98%. The nasal septum was swollen with discoloration of the nasal tip and surrounding area. Swelling extended to the right orbital region with ptosis of the right eye. He was diagnosed with NSA and periorbital cellulitis. Blood cultures and septic markers were obtained. While awaiting a CT scan in the ED, he vomited twice. He was initially started on intravenous ceftriaxone and paracetamol.

**Figure 1:**
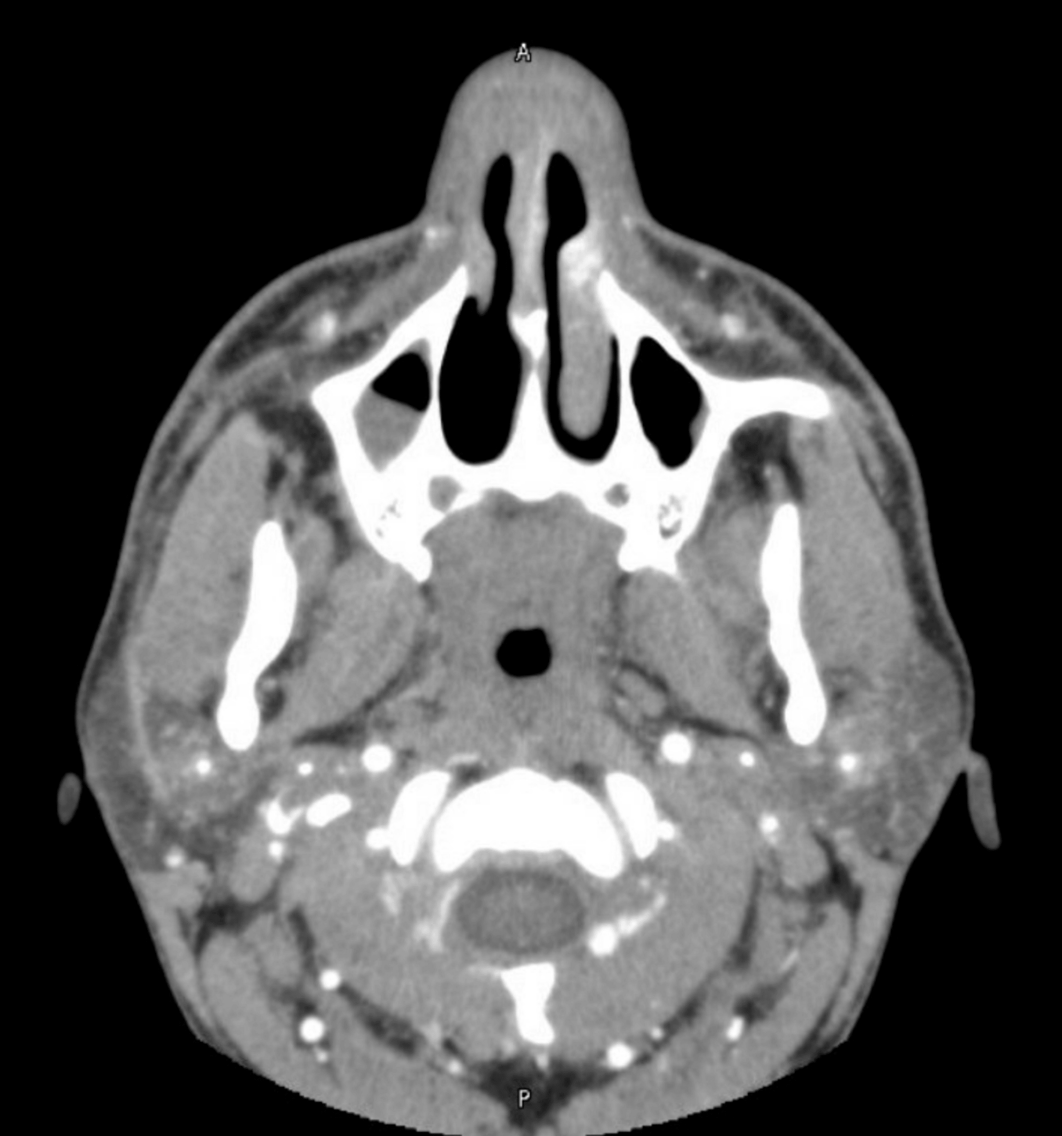
CT scan showing NSA NSA, nasal septal abscess

**Figure 2:**
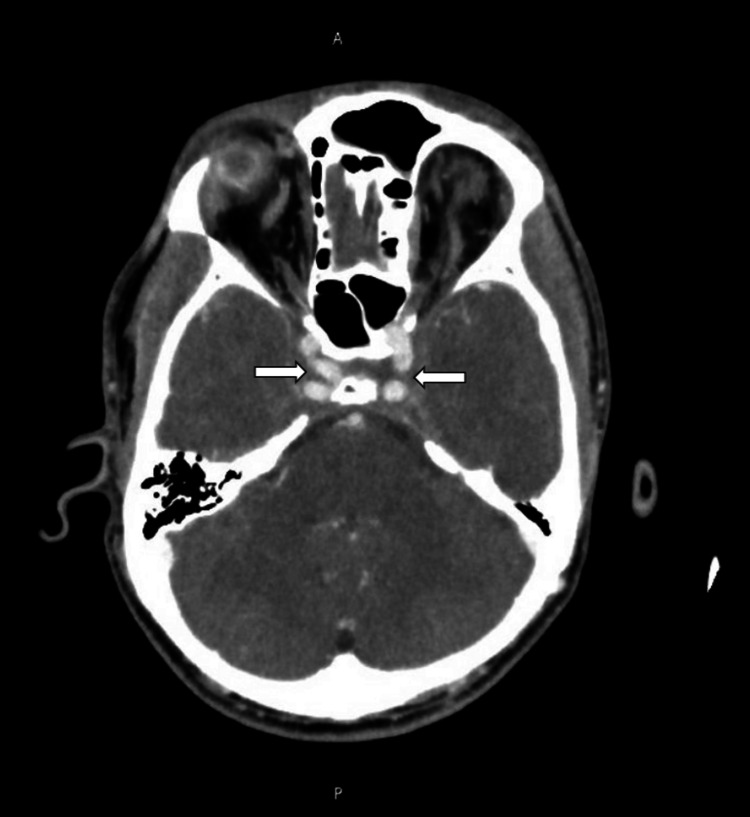
CT scan showing bilateral cavernous sinus thrombosis (white arrows)

A CT of the head, face, and sinuses revealed an NSA with right periorbital cellulitis and bilateral cavernous sinus thrombosis (Figure 1, Figure 2). The patient was admitted to the ICU. Anticoagulation was deferred to minimize the risk of bleeding in the setting of septic sinus thrombosis. Antibiotics were escalated to meropenem and vancomycin.

**Figure 3:**
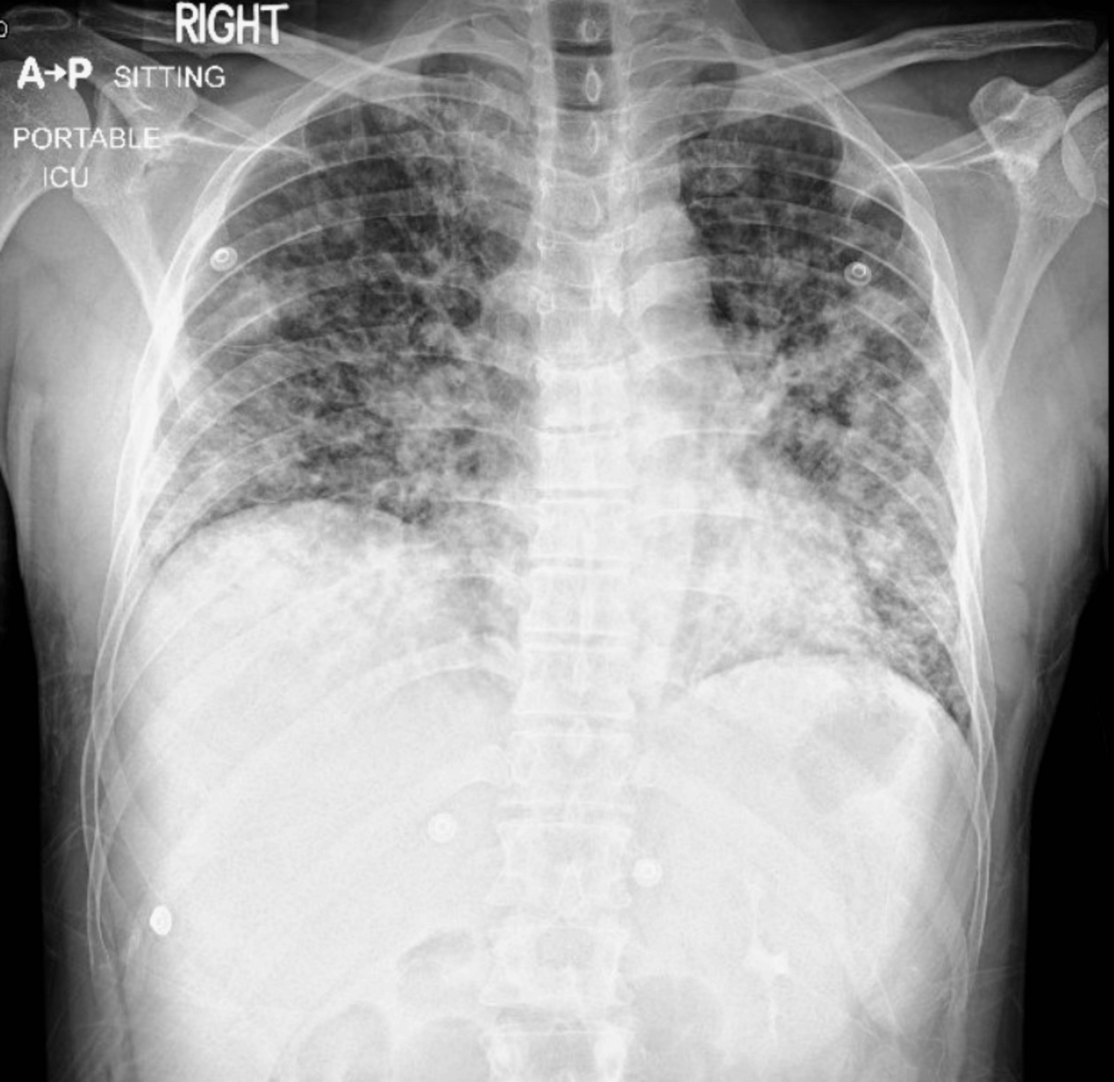
Chest X-ray showing bilateral lung infiltrates

On day 2, the patient developed tachycardia (132 beats/min), tachypnea (30-36 breaths/min), and fever (38.9 °C). He was placed on a high-flow nasal cannula; however, respiratory distress did not improve. Chest X-ray demonstrated diffuse bilateral infiltrates (Figure 3).

The patient was subsequently intubated and managed with lung-protective ventilatory strategies. Examination revealed delayed capillary refill, leukocytosis, and thrombocytopenia. Sepsis markers were elevated, and renal function parameters were deranged (Table 1).

**Table 1:** Septic markers and renal function parameters throughout the surgical ICU admission BUN, blood urea nitrogen; CRP, C-reactive protein; PCT, procalcitonin

Admission day	White blood cell (×10³/µL)	Platelet (×10³/µL)	CRP (mg/L)	PCT (ng/mL)	Serum creatinine (µmol/L)	BUN (mmol/L)	Serum lactate (mmol/L)	P/F ratio (mmHg)	Urine output (mL/hr)
1	8.5	167	325	12.9	72	4.9	7.4	N/A	110
2	2.9	68	539.7	N/A	76	6.1	2.6	73	20-25
3	11.1	51	649	48.1	108	14.7	2.2	84	20-25
4	21.2	33	643	74.1	277	22.5	1.8	102	40
5	29.1	45	476	N/A	393	33.1	2.1	241	80
6	26.3	45	472	>100	439	42.9	1.4	296	100-150
7	29.8	63	420	N/A	430	54.5	1.5	261	100-150
8	32.1	112	289	N/A	362	65.7	1.1	268	100-150
9	31.8	136	223	N/A	374	61	1	243	100-150
10	36.9	204	251	63.7	360	48.3	1	268	100-150
11	38.2	206	265	N/A	312	56.5	1.3	227	100-150
12	31.9	238	163	20.6	335	36.1	1.1	276	100-150
13	28.5	323	105	N/A	249	48.7	1	283	100-150
14	24.2	364	76.3	N/A	286	28.3	1	264	100-150
15	29.6	532	72.7	5.29	174	43.3	1.2	254	100-150
16	24.2	498	100.1	N/A	251	30.3	1.2	31	100-150
17	24.5	615	68.7	1.6	177	27.9	1	302	100-150
Reference range	4-11 ×10³/µL	150-450 ×10³/µL	Up to 10 mg/L	<0.05 ng/mL	44-97 µmol/L	7-20 mmol/L	0.5-2.2 mmol/L	>400 mmHg	0.5-1.5 mL/hr

The patient’s urine output was decreased (20-25 mL/hour), and his blood pressure dropped to 84/58 mmHg. Advanced hemodynamic monitoring was initiated via central line and PiCCO^®^ (pulse index continuous cardiac output) catheter insertion. Vasopressor support with noradrenaline and vasopressin, along with hydrocortisone, was required to maintain vital parameters.

He underwent incision and drainage of the nasal septal collection with washout under local anesthesia. Cultures of the nasal pus and blood grew MRSA, and antibiotic therapy was continued with meropenem, vancomycin, and clindamycin. Despite treatment, he remained in TSS, acute kidney injury (AKI), and severe acute respiratory distress syndrome (ARDS).

Given ongoing TSS, IVIGs were administered to neutralize bacterial superantigens and mitigate the cytokine storm. IVIG was given at 1 g/kg on the first day, followed by 0.5 g/kg/day for two subsequent days. Renal function worsened, and the patient became oliguric, necessitating continuous renal replacement therapy (CRRT). Echocardiography revealed severe left ventricular dysfunction with an ejection fraction of 27%, attributed to septic cardiomyopathy, and milrinone was added to the management plan.

**Figure 4:**
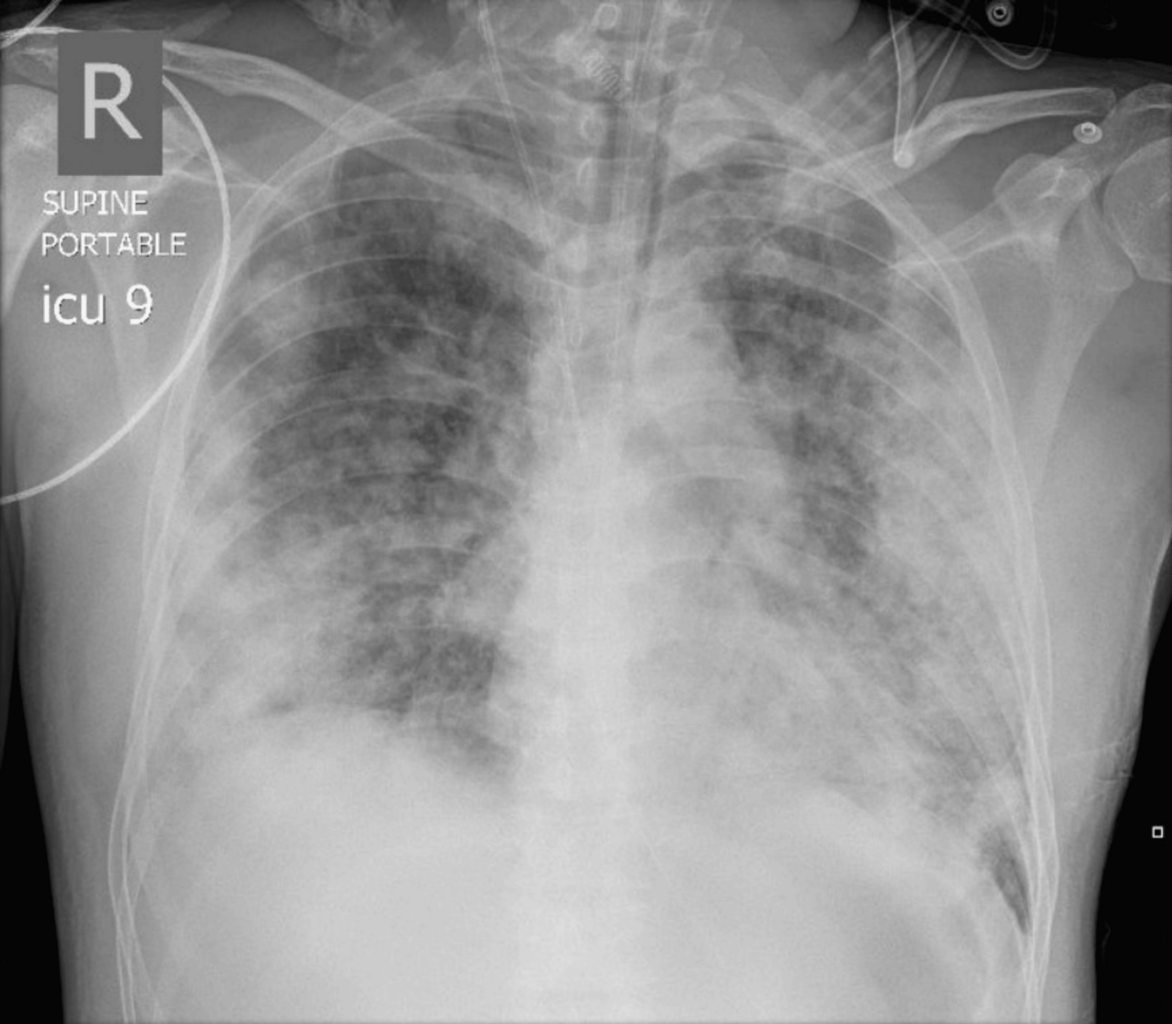
Chest X-ray showing multiple cavities

The patient remained on lung-protective ventilation for severe ARDS, with a P/F ratio of 73. He was managed in the prone position starting on day 4, resulting in improved P/F ratio and oxygen saturation (Table 1). Renal function stabilized, urine output improved, CRRT was discontinued, and furosemide was initiated. His respiratory status continued to improve, with the P/F ratio reaching 296 (Table 1). On day 6, a chest X-ray revealed multiple cavities (Figure 4).

**Figure 5:**
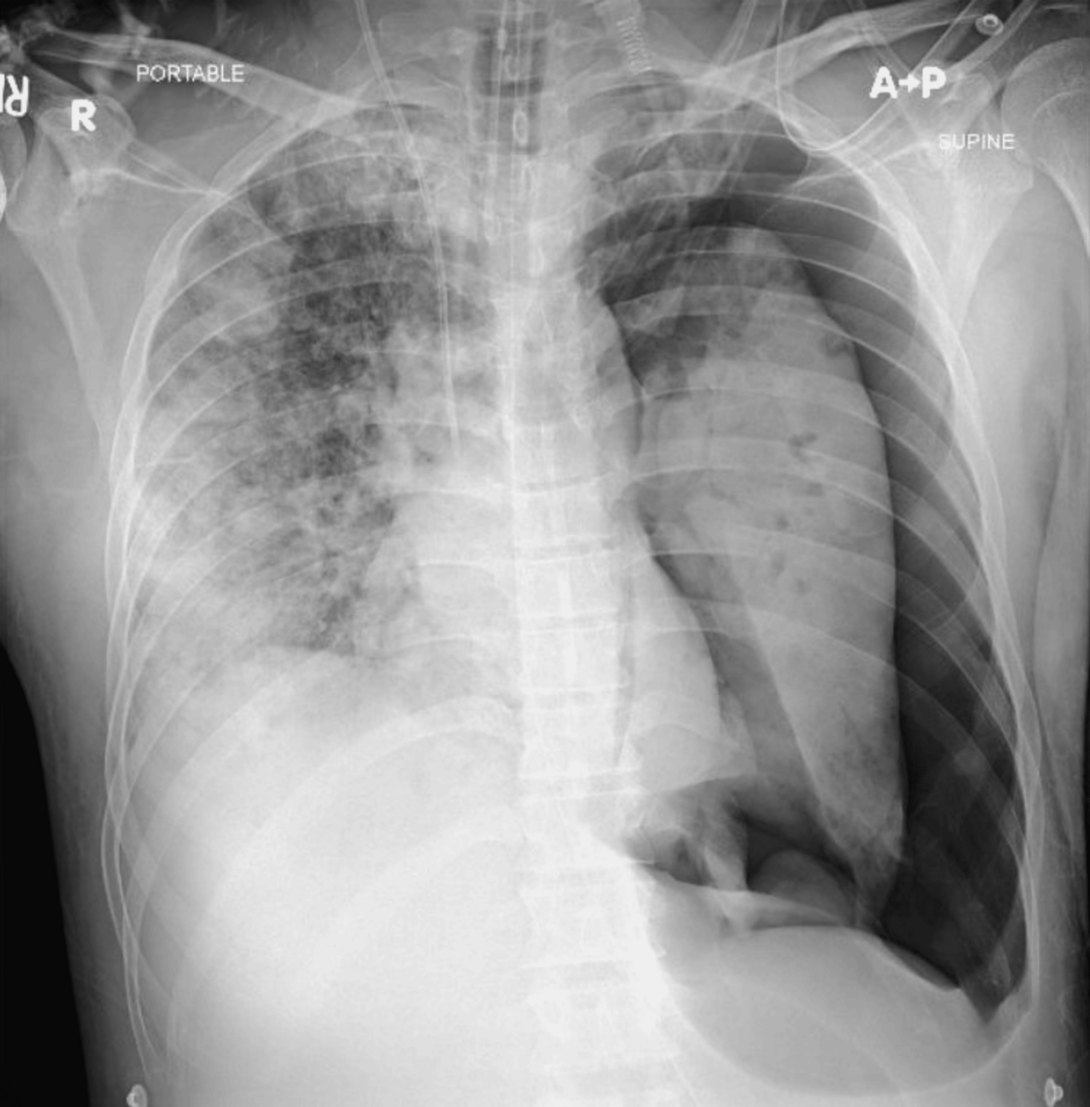
Chest X-ray showing tension pneumothorax

The patient suddenly desaturated to 80%, became tachypneic (RR 30-38 breaths/min), and exhibited decreased air entry on the left side. An urgent chest X-ray revealed a tension pneumothorax (Figure 5).

A left chest drain was immediately inserted, resulting in improvement of oxygen saturation to 97-98%. Organ dysfunction gradually improved, and urine output increased to 100-150 mL/hour. Furosemide was discontinued, and vasopressors were stopped by day 8.

The patient became responsive to external stimuli and obeyed commands but exhibited severe generalized muscular weakness. Despite a trial of weaning from mechanical ventilation under lighter sedation, he was unsuccessful. Electromyography confirmed a critical illness-associated neuromuscular abnormality, and he continued physiotherapy.

On day 19, a percutaneous dilatational tracheostomy was performed. All antibiotics were discontinued on day 20. Muscular strength gradually improved, and the patient was successfully weaned from the ventilator and managed via tracheal cannula by day 29. He was fully awake, able to take an oral diet, and transferred to the medical ward on day 32. Tracheal decannulation was performed on day 34, and the patient was discharged home on day 40 with outpatient follow-up. At three months, he was clinically well, with only mild cognitive impairment noted.

## Discussion

NSA is a rare clinical entity in adults but can result in life-threatening intracranial and cosmetic complications [5]. NSA is defined as the accumulation of pus between the bony and cartilaginous portions of the nasal septum and the mucoperiosteum. Beck classified NSA according to etiology into three categories: (i) traumatic; (ii) secondary to dental or sinus infections; and (iii) idiopathic, where the cause is unclear. In our patient, habitual nasal picking likely caused mucosal trauma, predisposing him to infection and subsequent NSA [6].

Sogebi and Oyewole identified older age, prolonged duration of symptoms, and a positive culture from nasal septal collection as risk factors for NSA [4]. Diabetes mellitus (DM) has also been increasingly reported as a risk factor, with differences in the causative organisms between patients with and without DM [7]. *Klebsiella pneumoniae *is the most common causative organism in diabetic patients, whereas MSSA and MRSA are most common in nondiabetic patients [7].

Reported complications of NSA include extension to periorbital cellulitis, regional spread to the brain causing meningitis or brain abscess, and cavernous sinus thrombosis, all of which can be fatal [8]. Our patient developed MRSA bacteremia secondary to MRSA NSA, which progressed to TSS and MODS. These complications have not been previously reported in the literature.

To the best of the authors’ knowledge, MRSA NSA and bacteremia causing TSS, complicated by AKI requiring hemodialysis, ARDS, and critical illness neuromuscular abnormality necessitating tracheostomy, have not been documented. MRSA bacteremia can progress to necrotizing pneumonia originating from septic foci [9]. Similar to our patient, individuals with MRSA or MSSA necrotizing pneumonia are at higher risk of developing tension pneumothorax, as previously reported [10,11].

## Conclusions

MRSA NSA can progress to life-threatening TSS with MODS and necrotizing pneumonia. MRSA necrotizing pneumonia may further complicate into tension pneumothorax during ventilator weaning, necessitating emergency decompression. Prompt incision and drainage with appropriate source control is essential to prevent local and regional complications in NSA patients. In cases of MRSA NSA, clinicians should maintain a high index of suspicion for bacteremia and TSS.
